# Morbidity of Rescued Wild Birds by Admission Causes in the Republic of Korea

**DOI:** 10.3390/ani14142071

**Published:** 2024-07-15

**Authors:** Haerin Rhim, Jooho Gahng, Geonwoo Baek, Myeongsu Kim, Jae-Ik Han

**Affiliations:** 1 Laboratory of Wildlife Medicine, College of Veterinary Medicine, Jeonbuk National University, Iksan 54596, Republic of Korea; helen814@hanmail.net (H.R.); zoology@kakao.com (J.G.); dhthflfkxpf@naver.com (G.B.);; 2 Jeonbuk Wildlife Center, Jeonbuk National University, Iksan 54596, Republic of Korea

**Keywords:** wildlife, diagnosis, admission, inflammation, trauma, infection, rehabilitation, rescue

## Abstract

**Simple Summary:**

Wild birds are at risk of death owing to human activities, including indirect human threats, such as environmental pollution, habitat destruction, and climate change. This study was conducted to analyze the records of birds rescued by the Jeonbuk Wildlife Center in the Republic of Korea to identify the problems and trends in terms of injury and disease that most affect free-living wild birds. From January 2019 to February 2021, medical records, including species, date, and location of rescue, cause of hospitalization, diagnosis, and treatment records, were obtained for all admitted birds. The analysis included 1464 birds belonging to 106 species. The main admitted causes were being orphaned, window collisions, and trauma from unknown reasons. Inflammatory conditions were predominant in diagnoses mainly caused by trauma and infection. The most frequent diagnoses were dehydration, integumentary injuries, and neurological injuries. The inflammation-related diagnosis accounted for 82% of all diagnoses.

**Abstract:**

Insufficient reports are available on what clinical and pathological conditions are observed in rescued free-living wild birds. This study investigated recent diagnoses of admitted wild birds based on admission causes in a southwestern area of South Korea over the past 2 years. A retrospective study was conducted on 1464 birds rescued from 2019 to February 2021. Overall, 12 admission subcategories were classified, and the diagnoses identified for each cause were analyzed. The three most frequently observed categories, general, integumentary, and musculoskeletal, each accounted for 20% of the total diagnoses. Trauma accounted for 71.4% of all diagnoses, and 81.5% featured inflammatory conditions, primarily due to trauma or infection. The proportion of birds that presented inflammatory conditions was much greater than the proportion of birds that were admitted due to trauma-related causes. This was because inflammatory diseases were identified at a high frequency, even from nontraumatic admission causes, and inflammatory conditions were not easily revealed. Suspecting an inflammatory condition in most rescued birds is advisable.

## 1. Introduction

As is already known, free-living wildlife has been under severe threat in the short and long term as human living areas have expanded and economic activities have flourished [1]. In particular, considerable declines have taken place, with up to 30% of bird species functionally extinct or vulnerable to extinction [2,3]. Not only have indirect effects, such as environmental changes, affected avian populations, but human activities have also directly caused serious losses to bird populations [4,5]. The number of animals admitted to our wildlife center has increased every year, and most of those admitted have been victims of human activity. Furthermore, as the territory shared between humans and wildlife has grown, the risk of zoonotic infection has increased. Numerous reports have reported that 70% of diseases that infect humans are of animal origin, and 75% of newly emerging infectious diseases are zoonotic [6,7,8,9]. The concept of One Health, in which human, animal, and environmental health are connected, has become widely accepted [10]. These issues have drawn considerable attention during the recent global COVID-19 pandemic.

Along with international responses to climate change and zoonotic diseases, many species have been designated as protected, and many wildlife rehabilitation centers and sanctuaries have been created. In Korea, since 2009, wildlife centers have been established in each province, operated sequentially with governmental aid. These centers play an important role in monitoring ecology and disease in wildlife, along with providing medical care [11,12]. Although a few studies have been conducted on admission causes to a wildlife center in Korea [13], little analysis has been performed on common morbidities and detailed diagnoses. If a rescued animal has no obvious injury, the prevalence of the disease can vary greatly in relation to how much additional testing is performed. In addition, many wildlife-related investigations have focused on a few diseases that affect humans and livestock. In general, wildlife clinics have cost limitations that impede in-depth examination. Although resources are minimal, more discoveries are being made with tests that are now available at a lower cost than in the past.

This study focused on birds because they tend not to be tested as often as mammals. This study covered information on admitted birds, the causes of admission, and clinical findings or diagnoses. Causes of admission by species were also investigated. As updated diagnostic tests provided a precise diagnosis in some cases, the latest trends in wild birds admitted in the past 2 years were summarized. Because many birds were found to have inflammation during inspections, this analysis addressed the causes of this and what we require for proper detection. We hope that this retrospective study will help wildlife experts to update their information and serve as a basis for further research in the future.

## 2. Materials and Methods

### 2.1. Data Collection

The records of all birds admitted to the Jeonbuk Wildlife Center from January 2019 to February 2021 were reviewed and analyzed. Out of a total of 2152 admitted wild animals, all 1464 birds (68%) were included in this study. Carcasses were not admitted to the center, nor included in this study. Collected information included species, age, location, admission cause, body weight, and medical records.

### 2.2. Admission Cause

The causes of admission were broadly divided into natural incidents, anthropogenic incidents, and others. These major categories were divided into 12 subcategories and 29 specific causes. Causes were classified according to the bird’s history, clinical signs, the geography and location of the rescue site, and clinical examinations. No individuals had their admission cause changed due to necropsy findings.

Traumatic causes included the following: injured orphans, being attacked, collisions, being injured by human-made structures, traffic accidents, poaching, and birds having experienced trauma for an unknown reason. The causes of admission causing inflammation included the following: injured orphans, being attacked, collisions, being injured by human-made structures, traffic accidents, poaching, birds having experienced trauma for an unknown reason, infections, and toxicosis.

(1) Natural causes
  A. Orphaned comprised two divisions: injured and congenital abnormality. This covered young birds that required rescue owing to injury or lack of vitality.
  B. Malnutrition included emaciated, weakened, or exhausted birds.
  C. Infection by viruses, bacteria, or parasites. In the case of bacterial infection, only those with bacteremia or sepsis were counted, and local infections were counted as another primary cause, such as trauma from an unknown cause. Because parasite infections are common in wildlife, only cases with marked symptoms as the main problem were counted.
  D. Attacked by other avian species, including their predators, as witnessed by the discoverer.

(2) Anthropogenic causes
  E. Orphaned included birds who were healthy and thus would not have required rescue but had been abducted by their discoverer or whose nest had to be removed.
  F. Attacked by cat or dog.
  G. Collision with either a window or a power line. Even if the person who submitted the bird for rescue did not observe the bird striking a window, it was considered a window collision if the bird was found next to a building bearing signs of collision or with no other presumed causes.
  H. Trapped indoors included birds who entered a building or poultry farm and could not find the exit. Those who bred their offspring on rooftops were also counted because the chicks could not leave owing to the structure of the building.
  I. Injured by human-made structures, including buildings. In this category, included birds were trapped in mouse glue traps, caught in the tangle of a string or fishing line, trapped in a net, stranded in a hole or canal used for agriculture, injured by a fishing hook, stuck between windows or signboards, injured by a mower, soaked in oil or contaminated, or roughly landed on asphalt.
  J. Traffic accident: collision with a vehicle.
  K. Poaching: injured by gunshots and body-hold traps.
  L. Toxicosis: because not all suspected cases were examined for chemicals, tentative diagnosis was also included based on the circumstances at the rescue site, physical examination, basic blood examination, an X-ray, and treatment response.

(3) Others
  M. Unknown: cases that did not fit into natural or anthropogenic causes were sent to other providers. These included cases of trauma from unknown causes and undiagnosed cases that had no identified abnormality or cases where no reason for abnormality could be found except trauma.
  N. Other: birds confiscated owing to legal protections.

Overlapping causes were classified according to the primary reason for the rescue.

### 2.3. Diagnosis

Diagnoses were based on the identifications made in various clinical and postmortem examinations. Unless the animal was dead on arrival, required emergency attention, or had clear reasons for euthanasia, we generally performed a physical examination, a fecal examination, an X-ray, and blood examinations, including manual complete blood count (CBC) and clinical biochemistry panels. Additional tests were conducted as needed: cytology, microbial cultures, antimicrobial susceptibility tests, molecular diagnosis, blood gas analysis, ultrasonography, computed tomography (CT), magnetic resonance imaging, ocular slit lamp microscopy, electroretinography, protein electrophoresis, auditory brainstem response tests, necropsy, and histopathological examination. In the case of multiple diagnoses, all were included in the analysis. All diagnoses included this study were from original conditions.

The classification was made as follows: general (clinical symptoms that could not be included in one system), infection, circulatory, digestive, integumentary, musculoskeletal, neurologic, sensory, respiratory, and urinary systems. The general category comprised dehydration, hyper-/hypothermia, shock, and cachexia.

## 3. Results

Overall, 1464 individuals belonging to 106 species, 41 families, and 17 orders were diagnosed (Table S1). In order to reduce redundancy, only common names are used in the main text, and species information is described in Table S1. The 10 most frequently rescued species accounted for 60.0% of the total diagnosed population. Birds of prey, including Accipitriformes, Falconiformes, and Strigiformes, comprised the largest category, with 382 individuals (26.1%), and 376 water birds were present, including Anseriformes, Charadriiformes, Gaciiformes, Gruiformes, Pelecaniformes, and Podicipediformes (25.7%). Passeriformes included the largest number of species and populations in a single order, with 37 species and 359 individuals (24.5%), followed by Columbiformes (242, 16.5%), Anseriformes (216, 14.8%), Strigiformes (165, 11.3%), Falconiformes (135, 9.2%), and Pelecaniformes (115, 7.9%). At the family level, Columbidae (16.5%) were the most common, followed by Anatidae (14.8%), Strigidae (11.3%), Falconidae (9.2%), Ardeidae (7.6%), and Corvidae (7.4%). Nestlings and fledglings accounted for 561 of the diagnosed birds (38.3%), and 903 birds (61.7%) were juveniles or adults. Approximately 61% of the birds were admitted between the May and August of the study years.

The causes of admission with age information are given as follows (Table 1; causes by month are given in Table S2). Natural causes accounted for 219 (15.0%) cases, 1016 (69.4%) birds were admitted owing to anthropogenic causes, and 229 birds were categorized as others (15.6%). The largest category of causes was orphans who did not need to be rescued (23.5%) and window collisions (20.9%). Trauma from unknown reasons (9.8%), one of the major causes, was followed by trapped in a building/farm (6.2%), exhaustion/emaciation (6%), undiagnosed (5.5%), and hit by a car/train (4.9%). Trauma affected 750 birds, accounting for 51.2% of cases. Inflammatory causes, including trauma, accounted for 57% of cases, at 835 birds (Table S3).

**Figure 1:**
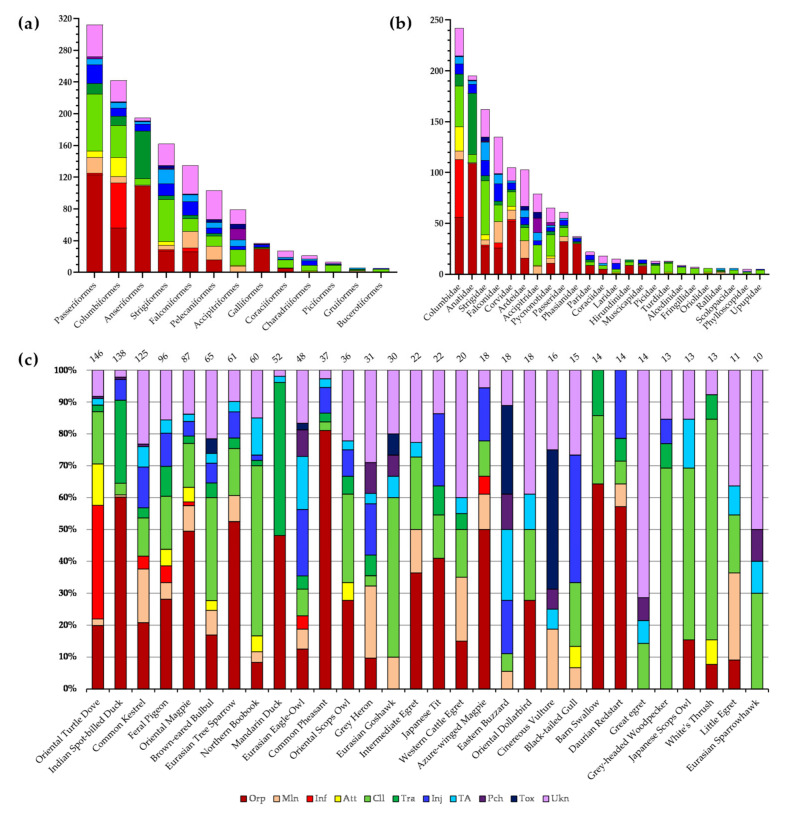
Causes of admission by (**a**) order, (**b**) family, and (**c**) species. For convenience, in (**b**), only families with more than 5 birds are included, and in (**c**), all species are set at 100%, and each cause is depicted as a ratio. Total populations are indicated on each of the columns (Orp: orphaned, Inf: infection, Mln: malnutrition, Att: attacked, Cll: collision, Trp: trapped indoors, Inj: injured by human-made structures, TA: traffic accident, Pch: poaching, Tox: toxicosis, Ukn: unknown).

The causes of admission by species, family, and order are presented in Figure 1. At the family level, Columbidae had the highest rate of infections and being attacked among all families. Anatidae were admitted mostly owing to being orphaned or being trapped indoors. The most common admission reason for Strigidae was collision, almost all of which were window collisions. Traffic accidents represented the largest percentage of causes across all families. We did not rescue any orphans from Accipitridae, and they had a high rate of being poached.

The cause of admission of the top 30 species admitted is shown at the species level (Table 2). Oriental Turtle Doves had an overwhelmingly high rate of rescues due to infections (35.6%, 52/146) and were also the species with the highest rate of rescue from attacks by other animals (13%, 19/146). About 60% of Indian Spot-Billed Ducks were rescued as orphans, and, along with Mandarin Ducks, the rate of collisions was less than 4%, which was very low relative to other species. The rate of malnutrition among Common Kestrels was higher than that of other species at 16.8% (21/125), and the rate of those injured by human-made structures was also relatively high at 12.8% (16/125). The main cause of admission for Oriental Magpies and Eurasian Tree Sparrows was being orphaned (approximately 50%). For Brown-Eared Bulbuls, the main reason for rescue was collision (32.3%, 21/65) rather than being orphaned. The Northern Boobook, along with the Eurasian Goshawk, had the highest collision rate among the top 20 species, with more than 50% experiencing window collisions (32/60 and 15/30 each). Most Common Pheasants were brought in as orphans (81.8%). The collision rate was similar to that of the orphans in Oriental Scops Owls (27.8%, 10/36 each). In Grey Herons, malnutrition was the most frequent cause of admission (22.6%, 7/31), excluding unknown causes.

The clinical findings and diagnoses of the examined birds by system were analyzed in order of frequency, regardless of the cause of admission (Table 3). The most common findings relate to the integumentary system (20.2%), followed by the musculoskeletal (19.4%) and general category (19.2%). The nervous system accounted for 8.8% of cases, and infection accounted for 7.9%. The sensory category accounted for 7.5% of cases and mostly consisted of ocular disorders. After categorizing each finding as to whether it was caused by trauma or accompanied inflammation, 3394 diagnoses were counted as traumatic (71.4%, 1359 non-traumatic) and 3874 were counted as inflammatory diagnoses (81.5%, 879 non-inflammatory).

The most frequent individual finding was moderate to severe dehydration, which was identified in 36.5% (534/1464) of admitted birds. Subcutaneous, skin, and muscle damage also dominated diagnoses, found in more than 20.5% (300/1464) of birds. The wings were the most vulnerable body parts to trauma, followed by the body and legs. One-fifth of birds (299/1464) were admitted with traumatic brain injury (TBI). Ocular trauma, commonly accompanied by TBI, was observed in 111 individuals. Traumatic uveitis, corneal abrasions or ulcers, hyphema, preretinal fibrosis, vitreous hemorrhage, retinal tear or edema, and anterior vitreous prolapse were all commonly diagnosed. Retinal laceration or detachment was found in 27% (30/111) of individuals with ocular trauma. Bullous keratopathy was identified in two cases, both of which featured oral plaque owing to an infection with a *Trichomonas* species and ocular exudate. This occurred as the scabs of the cornea were removed from the ocular exudate. Glaucoma was found in one individual with traumatic uveitis. About 14% [18/129(111 + 18)] of ocular lesions were infectious or senile lesions not due to trauma.

Kidney abnormalities (11.2%, 164/1464) also ranked high, including acute kidney injury, mainly resulting from dehydration. Hepatitis was one of the most frequent findings during necropsy, confirmed in 60 birds, and liver rupture, necrosis, and abscess were found in that order. Inflammation and hemorrhaging of the gastrointestinal tract were observed in 52 cases, and ulcers or perforations were confirmed in 15 cases. In some cases, this was caused by the intervention of foreign objects, such as fishing hooks or bullets, but, more often, the cause was unknown.

Moderate to severe anemia was confirmed in 8.9% of birds (131/1464) with CBC, and severe emaciation and malnutrition were found in 7.4% of cases. Leukocytosis was found in 60 birds, among which 5 had severe systemic inflammation and bacteremia. Elevated white blood cell (WBC) count varied from 17 up to 160 × 10^3^/µL, and heterophilic inflammation was the most common. No actual cases of leukemia were found. *Staphylococcus* species, *Salmonella* species, and *Pasteurella multocida* were confirmed in samples from the infection site of birds with severe systemic inflammation. Leukopenia was confirmed in five birds through blood smear examination, and this was mainly owing to acute severe inflammatory reactions.

Protozoa belonging to the *Trichomonas*, *Eimeria*, and *Isospora* were found in 22 birds. These were largely in the form of typical plaques of trichomoniasis in the oral cavity; however, in some individuals, severe infection spread beyond the oral mucosa to the esophagus, mandible, maxilla, and skull. *Haemoproteus* or *Plasmodium* infection was confirmed in 50 birds through blood smear examination, and the rate of *Haemoproteus* species infection was seven times higher than that of *Plasmodium* species, as confirmed by molecular diagnosis.

A local bacterial infection that formed purulent exudate or abscesses was confirmed in 3.8% (55/1464) of cases. *Enterococcus faecalis*, *Enterobacter* species, *Staphylococcus* species, including *S. aureus*, *S. sciuri*, *P. multocida*, and *Escherichia coli* were frequently identified bacteria in the first sample cultures before treatment. Other confirmed bacteria included Bacillus species, including *B. cereus*, *Klebsiella pneumoniae*, *Proteus mirabilis*, *Pseudomonas aeruginosa*, *Streptococcus* species, *Corynebacterium* species, *Shigella* species, *Aeromonas* species, and *Vibrio* species. In open fractures, infection by the Enterobacteriaceae family was the most common (34%), followed by *Enterococcus* species infection (21%). With respect to superficial injuries instead of open fractures or deep infections, the genera *Enterococcus* and *Staphylococcus* were frequent, representing 30% of cases. Yeast infection was often confirmed through fecal examination, which was generally of a secondary proliferation owing to a primary cause. Infections with *Mycoplasma* species or *Campylobacter jejuni* were confirmed through infectious disease screening diagnostic tests performed with samples taken from individuals who underwent necropsy in 2019. Infection with *Mycoplasma* species was confirmed in 15 birds, including raptors and egrets, *C. jejuni* was found in 3 birds, and no species of the genus *Chlamydia* were detected. Because of accurate identification, the *Mycobacterium* species was confirmed to be *M. abscessus*, not *M. tuberculosis*.

Pulmonary injury and inflammation were detected in 5.5% (80/1464) of admitted birds, among which pulmonary hemorrhages and injury owing to trauma accounted for 67.5% (54/80). Pneumonia, abscesses, and granulomas resulting from bacteria or fungi were also occasionally identified during necropsy. Air sac rupture was confirmed in 24 birds, and airsacculitis was observed in 9 cases, confirmed as being due to bacterial or fungal infections. In two individuals, fibrinous changes in the air sacs were confirmed, but no infectious agent was identified.

### Morbidity by Admission Causes

A. Orphaned

Most diagnoses were of injured orphans, comprising 13.5% (54/400) of all orphans. Diagnoses commonly found in this group included dehydration (14.8%, 59/400), skin injury (12%, 48/400), fracture (9.3%, 37/400), exhaustion (5%), malnutrition (4.7%), severe ectoparasite infestation (4.6%), and metabolic bone disease (1.2%). Although tests could not be conducted owing to the very small size of most birds, severe electrolyte imbalance or acid–base imbalance was confirmed in half of the cases. The most common injury was hindlimb fracture, followed by spinal fracture, mostly owing to falling from the nest. Hepatitis, nephritis, and pneumonia were also confirmed in 10 birds (2.5%) who died at the center while being treated.

B. Malnutrition

Severe dehydration was observed in 90% (79/88) of cases, and severe emaciation was observed in 39.8% (35/88). Anemia was found in 30% (26/88) of cases. Elevated liver enzymes (22.7%) and hypoalbuminemia (15.9%) were also frequently found. Hypothermia (18.5%), elevated uric acid (16%), hyperkalemia (9%), shock (8.8%), and hypernatremia (6%) were also observed in many cases. Systemic inflammation, severe infestation of ectoparasites or hemosporidia, hepatitis, pneumonia, and campylobacter infection were confirmed in fewer than 10 individuals each. About 15% of individuals had systemic or local inflammation or protozoan infestation.

C. Infection

Viruses were the most common pathogen, at 77.3% (51/66), all of which were avian poxvirus, followed by parasites and bacteria. Excluding asymptomatic infections, 80% (8/10) of symptomatic parasite infections were *Trichomonas* species. Cutaneous masses around the face and beak caused by the poxvirus were identified for 80.4% (41/51) of cases, and those occurring on the legs and digits accounted for 35.3% (18/51) of cases. Skin lesions were also occasionally found on the wings. In protozoan infection, they were identified in plaques from the oral cavity or in feces. Dehydration (77.8%, 7/9) and malnutrition (55.6%, 5/9) were major clinical findings. Among bacteria- and parasite-infected birds, anemia (33.3%, 5/15) and leukocytosis (33.3%, 5/15) were common findings, with 20% (3/15) of individuals having WBC levels above 20 K/μL. Severe heterophilia and leukemoid reactions were noted in four cases, with levels above 50 × 10^3^/µL found through manual counting.

D. Attacked

Cat attacks were far more common than dog attacks, accounting for 77.5% (31/40) of attacks. Bite wounds on the body were observed the most, in 67.5% (27/40) of cases. Fractures were found in almost half of the birds. More than one-quarter of the birds were in shock. Wounds to the wing and legs accounted for 29% and 19% of wounds, respectively. The main bacteria identified at the bite site were *P. multocida*, *Neisseria zoodegmatis*, *Bacillus proteolyticus* or *B. wiedmannii*, *Staphylococcus* species, and *Streptococcus* species.

E. Collision

Almost every bird (96.8%, 306/316) rescued after a collision had hit a window, and 3.2% (10/316) collided with a power line. About 70% (214/306) of those that had crashed into a window showed symptoms of TBI. Half of these individuals had severe dehydration and/or bleeding from the nasal or oral cavity. Traumatic ocular injuries were confirmed in 60.1% (190/316) of all collision cases. Ocular trauma was particularly high in owls, which have a large head-to-eye ratio. Pulmonary abnormalities were identified in 29.7% (94/316) of cases, and most were pulmonary hemorrhages. Upon necropsy, pneumonia and lung abscesses were found in six cases. Fracture was seen in 49.1% (155/316) of birds. The most commonly affected bones were the humerus (11.5%), followed by the coracoid (9.9%), ulna (7.7%), radius (7.3%), synsacrum (6.4%), metacarpus (3.5%), clavicle (2.9%), keel (2.2%), thoracic vertebrae (2.2%), and scapula (2.2%), in that order. Hepatitis was confirmed in 20 birds (6.4%), and abnormalities, such as liver fibrosis, necrosis, and rupture, were confirmed in 15 cases (4.8%). Five individuals (1.6%) with severe hemorrhaging in the body cavity owing to a rupture of the internal organs, such as the liver, were present. Enteritis and nephritis were identified in five and four birds, respectively. Ulcers and perforations in the gastrointestinal tract from unknown reasons were also observed.

F. Trapped indoors

Common clinical findings in birds trapped indoors were dehydration, increased aspartate aminotransferase (AST) and creatine kinase (CK) levels, and hypoalbuminemia. They were usually directly released from the site, and the number of birds tested was very small.

G. Injured by human-made structures

Dehydration and exhaustion were prominent in 70.1% (73/104) of birds in this category. In the case of glue traps, severe exhaustion (84.6%, 88/104) and electrolyte imbalances (36.5%, 38/104) were common diagnoses on top of feather contamination (82.7%, 86/104). Birds weighing less than 60 g had a high mortality rate owing to severe exhaustion and dehydration. Occasionally, ruptured air sacs (7.3%) and skin abrasion or lacerations (7.3%) were found. Damage from a string or fishing line was found on the legs and wings with the same frequency. Wing tip syndrome was noticed in three cases (2.9%) in the distal wing, resulting in dry gangrene syndrome. Fishing hooks were confirmed with equal frequency when on the wing and when swallowed.

H. Traffic accident

Fractures were observed in 65.3% (47/72) of individuals in this category and were most common in the humerus, radius, and ulna, in that order. Spinal fractures (12.5%, 9/72) were also frequently diagnosed, and many had paraparesis owing to spinal injury. TBI was observed in 48.6% (35/72) of cases. Traumatic uveitis, corneal abrasions or ulcers, hyphema, preretinal fibrosis, retinal tear, cataracts, and subluxation or dislocation of the lens were found in approximately 30% of cases. Anemia was found in 20% of cases, and hepatitis was relatively high (10%) among all causes of admission.

I. Poaching

Gunshots and traps caused fractures in 90% (18/20) of birds. Gunshot wounds were most common in the wing (56.3%, 9/16), and the frequency of body and leg injuries was similar (38%, 6/16 each). Anemia and leukocytosis were confirmed in half of the poaching injuries, and four birds were rescued from shock. All four individuals caught in traps either had already had their legs or wings amputated or they needed to be amputated owing to interrupted blood flow and severe nerve injury. A foot was almost amputated in three individuals, and one was caught in a trap by their wing.

J. Toxicosis

Open-mouth breathing, increased oral mucus, and ataxia were the most common clinical symptoms of toxicosis. Increased AST was confirmed in 63.2% (12/19) of cases, and high CK levels were observed in 57.9% (11/19). More than half of the birds (11/19) were unable to stand. Gastrointestinal content was seen in most cases, and 36.8% (7/19) of birds retained food in the crop as well.

K. Unknown

The results of trauma from an unknown reason were similar to those of the diagnoses collected from window collisions and traffic accidents, including the most injured area and frequency of injury. When traumas of unknown cause were analyzed, integumentary injuries (64.4%, 145/225), fractures (59.1%, 133/225), and severe dehydration (49.8%, 112/225) were frequent, which was very similar to those caused by collisions. Hypoalbuminemia and anemia were found in about 20% of cases. About 10% of birds could not stand or fly after their injury from an unknown cause. Hepatitis was found in 8% of cases, and the rates of ocular trauma and cerebral hemorrhage were similar to those for other blunt trauma. Abscesses or granulomas of the liver, lung, and spleen were identified in fewer than 10 animals.

L. Other

All four birds in this category were temporarily fostered after being confiscated as illegally raised protected species and before transfer to a suitable organization. Coccidia infection was confirmed in all birds, and one had severe conjunctivitis and sinusitis suspected to be mycoplasmosis.

## 4. Discussion

This analysis examined the clinical symptoms and morbidities that rescued birds displayed upon admission, as assessed through a data-driven approach, not via empirical estimation. As many reports have noted, anthropogenic factors accounted for almost 70% of admissions [14,15]. Along with window collision, orphaned birds represented a large proportion of admitted birds, indicating considerable habitat overlap, which was also connected to a high risk of exposure to anthropogenic injuries. Trauma is the dominant cause of rescue worldwide, affecting 50%–90% of birds, consistent with our study [12,14,16,17,18,19,20]. The actual rate of inflammation confirmed through physical examination and basic diagnostic tests was much greater than the rate of trauma or inflammation-related causes of admission. This was because inflammatory diseases were found at a high frequency even in nontraumatic admission causes, and, in some cases, the inflammatory condition was not predictable.

Although the cause of rescue was classified according to the reporter’s statement, evaluation by rescue workers, and visual evaluation by a veterinarian after admission, the cause of rescue may have been be misclassified. This was confirmed in our study, as birds for which the cause of admission was changed after several tests were performed accounted for 2% of the total number of birds for which treatment was actually performed (among 921 birds, excluding those that were euthanized or died at admission). Skipping diagnostic tests can result in the misclassification of individuals as healthy or simply frail. Infection and disease have the highest risk of being underestimated. These may be misclassified as malnutrition or an unknown cause. Alternatively, they may be classified as having a visible trauma, with the underlying disease being missed. With more and more varied tests being performed, and on as many individuals as possible, a higher rate of diagnosis will be obtained, although practical limitations exist. Depending on how many tests were performed, the number of infections was either not counted at all or was very low [21]. Montsec (2016) noted that unidentified or masked cases with an infection owing to financial limitations would be classified as malnourished. Malnourishment was not a rare admission cause; however, a thorough examination should be performed on such animals, as many of them may have underlying diseases, such as severe inflammation or infection. Because we maintained our first admission causes, which were usually finalized within a few days, we also found unexpected diagnoses, such as protozoal infection from birds with malnutrition or hepatitis from birds who were unnecessarily rescued orphans. Nevertheless, the reason why the admission cause was not changed according to the necropsy findings was because the findings could not always be said to be the major cause of rescue. A case of bacterial pneumonia was also found in a Common Kestrel that was trapped in a window frame but died two days after hospitalization. Previous reports on birds in Korea have found infections in around 1% of the population [13,22]. A wide range of diverse infectious agents was found in this study, forming 4.5% of admission causes and 7.9% of diagnoses, which can increase more depending on additional tests.

Dehydration was the most frequent individual finding, although it was only counted when its signs were obvious. This is consistent with the literature that has found around 5% of wild animals rescued by wildlife centers to be dehydrated [23]. However, more than half of these birds rapidly normalized within a week, as dehydration can improve through hydration treatment and proper nutrition. Severe electrolyte and acid–base imbalances were commonly observed, and adequate fluid therapy is considered the easiest and most effective treatment at any wildlife clinic. Injury to the musculoskeletal and integumentary system was confirmed in more than half of individuals during the first physical examination. The most common fracture locations were similar to those previously reported in Korea [24,25,26]. Excluding head trauma, the wings were the most vulnerable to injury. TBI resulting from head trauma caused the overwhelming majority of neurological abnormalities. Ocular trauma was commonly found in birds with trauma in this study, as is frequently observed at other wildlife centers [27,28,29]. This was prominent in window collisions and traffic accidents, resulting from strong blunt impacts to the skull and ocular region.

Abnormal results from routine CBC were often found, such as anemia and leukocytosis. If manual counting is not applicable, packed cell volume (PCV), total solids, and smear examination are recommended to acquire minimal essential information. Blood loss, inflammation, and malnutrition are the most common causes of anemia in small animals. Although the cases reviewed in this study had various levels of severity, most cases showed a regenerative reaction to proper nutritional support and treatment. Even among the birds that had severe anemia, with a PCV below 20%, many showed rapid improvement within 1 or 2 weeks with appropriate treatment, without requiring blood transfusion. Inflammatory leukocytosis was often identified even in birds without trauma where an inflammatory response was not expected. Periodic blood examination is recommended, as an inflammatory response is frequently identified using general CBC and biochemistry tests. On top of WBC counts, continuous albumin and albumin/globulin ratios provide reliable information for understanding the current health condition and treatment response [30].

Various pathogenic, opportunistic, and antibiotic-resistant bacteria were identified at open fractures or in deep soft tissue damage. In a study conducted at our institution investigating bacterial flora collected before the initiation of dosing or treatment, the high prevalence of methicillin-resistant and multidrug-resistant bacteria was noted in wildlife [31,32]. A study of bacteria conducted in Spain found that antibiotic resistance was detected in the open fractures of wild birds, similarly to this study [33]. While antimicrobials have never been administered in the wild, finding resistant bacteria in the wild is not uncommon, suggesting that they have already spread in the environment within free-ranging and captive wildlife [34,35,36,37]. Birds can disseminate resistant bacteria to many locations much more widely than mammals, making this observation especially important [38]. Although surveys of rescued wildlife cannot represent the entire community of wildlife, continuous follow-up is nevertheless required as a useful indicator for the presence of antimicrobial-resistant bacteria present in the natural environment [39]. The rate of resistant bacteria in wild animals found in this study was lower than in companion animals; however, the absolute rate was not low [40]. For this reason, performing an antimicrobial sensitivity test where a condition does not improve with empirical antimicrobial treatment is necessary.

Poxvirus was the most common infectious agent, confirmed through molecular diagnosis in every suspected case. Typical dry cutaneous lesions in the head and digits were the most common features, as reported in many descriptions of avian poxvirus in Korea [41]. The condition of birds varied from bright and alert to severely debilitated, depending on the size and number of the nodules and how limited the birds’ food intake was. Diphtheritic or mixed forms were confirmed in two cases. Infections have been reported in more than 230 species worldwide; however, Oriental Turtle Doves accounted for 100% of cases in our study [42,43]. The case of one AIV-positive water bird was found not because of any neurologic signs but because of a window collision, implying that the possibility of wildlife bringing in infectious diseases should always be considered.

A previous study showed a high prevalence of hemosporidia in wild birds in Korea [44]. Most cases in native birds are nonpathogenic and asymptomatic and have not been thought of as a cause of rescues by wildlife centers [45]. This study featured two juvenile Eurasian Eagle-Owls that suffered severe hemosporidia and anemia (around 20% PCV). Apart from hemosporidia, no abnormalities were found, and the birds improved rapidly and were released after antimalarial treatment. This suggests that hemosporidia should be considered as a primary cause of rescue. Filariasis in avian species is mainly nonpathogenic and rarely reported in Korea [46]. Herein, it was identified incidentally in the peripheral blood of one Oriental Dollarbird.

Infection with *Mycoplasma* species was not rare in this study, and this supports the findings of another study where multiple pathogens were incidentally screened [47]. *Mycoplasma gallicepticum*, a representative pathogen of *Mycoplasma* infection in birds and the most important disease in poultry, has been observed in 56 wild avian species [48]. It is a frequent cause of conjunctivitis in small birds belonging to the Passeriformes [49,50]. Mycobacteriosis, including that caused by *M. avium*, which is the most common bacterial species in avian species, was reported in six cases of free-living raptors in the United States [51]. In our study, *Mycobacterium abscessus*, a non-tuberculous mycobacterium, was observed in a Eurasian Eagle-Owl [52]. *Salmonella* species were identified in a few Feral Pigeons with septic arthritis or severe systemic inflammation. Considering the prevalence of around 1%–3% that has been reported in wild birds in the United States and Trinidad, the frequency of the detection of this pathogen is likely to increase if more individuals are tested [53,54,55,56].

Pulmonary and hepatic abnormalities were revealed more often during necropsy than in premortal examination. Pulmonary hemorrhage owing to trauma was the most common observation, and hepatitis was found in many birds through necropsy and histopathology. Hepatitis was also discovered in birds that had been classified as having an unknown admission cause, leading to infection being thought to be the major reason for rescue. However, it was also found in other cases, such as window collisions or those isolated indoors, making it difficult to consider it as the primary reason for admission. In the future, conducting investigations of highly likely infectious diseases targeting individuals with hepatitis will be necessary. Bacterial abscesses, fungal granulomas, and severe inflammation of the lungs were also occasionally identified. Fungal infections of *Aspergillus* species have been commonly reported in wild birds. We confirmed one bird out of two cases with granulomas confirmed through CT that completed long-term treatment ending in a successful release. In addition to aspergillosis, a case of fungal infection of *Epicoccum* species was identified, although this was not during the period of this study [57].

For cases of suspected poisoning, although we included some birds who were not definitely diagnosed, we only included those subjects that were strongly considered to be experiencing toxicosis, such as passerine birds that fell ill en masse after eating seeds or birds of prey that were confirmed to collapse a few hours after eating a corpse. However, witnessed cases are rare in many rescued wild animals, and detecting an exact cause is always challenging as they do not show toxin-specific clinical symptoms. As with infection, toxicosis was likely to be underestimated and misclassified as having an unknown cause. According to one U.S. wildlife diagnostic laboratory, poisoning was the most common cause for the referral of carcasses, even more than trauma [58]. Similarly, in one study conducted in Korea, 40% of dead bird carcasses were identified as having died owing to acute poisoning [59]. These dead birds were waterfowl and geese, and both studies found phosphamidon as the most common agent. Together with monocrotopus, organophosphorus pesticides were also frequently identified. A domestic report indicates that phosphamidon was detected in Eurasian Goshawks suspected to have died from poisoning, and carbamate poisoning has also been detected in group deaths of great white-fronted geese [22]. No confirmed cases of lead poisoning were found in our study; however, according to another study in Korea, 3 out of 27 waterfowl carcasses showed very high lead concentrations, and 17.4% of all carcasses with various causes of death had sufficient Pb residues [60].

In some cases, a definitive diagnosis could not be established due to indeterminate causes. Given that wild animals often lack a detailed history, it was possible that they might have experienced mild symptoms and subsequently recovered or that they might have been exposed to toxins or had undiagnosed infectious diseases. However, when clinical symptoms improved and the animal was deemed ready for release, it was ideal to return them to their natural habitat as soon as possible, even if a definitive diagnosis had not been made. Thus, treatment outcomes rely heavily on rapid diagnosis and intervention through various differential diagnoses based on accumulated data, as discussed in this study. Considering the high incidence of inflammatory conditions observed in this study, it is advisable to consider inflammation as a likely factor in most rescued birds.

Our wildlife center forms an important bridge not only to the treatment of injured individuals for a humanitarian purpose but also to understanding the population, ecology, and diseases of wild animals. We have accumulated a wide range of data on rescued animals, including information from what kind of request is received to the species found, the frequency, region, and topography, the time, rescue causes, the diagnosis, and treatment results. In particular, diseases occurring in wild animals are also indicators of diseases that can pass to humans and livestock, and diseases mediated by humans and livestock can cause damage to wild animals. Because a limitation is observed in conducting individual research on various diseases, data from wildlife centers play an important role as a window to part of nature [61,62]. These data also play an important role in species conservation by tracking and managing the population of species that has decreased in number through disease detection [63]. This study was limited by its short study period; therefore, future studies should seek to document the exact cause and diseases of wild animals through various diagnostic tests over a long period. Additionally, all records were thoroughly reviewed to ensure that only diagnoses resulting from the original problem were included. However, we could not fully rule out the possibility that acquired abnormalities were included in the necropsy results. The data generated through this work can provide insights from various perspectives, from real-time risks to wild bird populations, including existing pathogens, the development of appropriate management preparations, and the creation of appropriate ecological policies. We hope that the results of this study will be used to assist wildlife professionals with successful rehabilitation in the short term through accurate preparation while efficiently managing future intake in the long term.

## 5. Conclusions

Anthropogenic causes, characterized by window collisions and orphans that did not require rescue, were prominent at 69.4% of intake over the course of 2 years, and traumatic causes were identified at 71.4% of all diagnoses. Considering trauma and infection together, inflammatory diseases accounted for 81.5% of the diagnoses. The detection rate of infectious pathogens and lesions was higher than in previous reports, indicating the necessity of continuous investigation.

## Figures and Tables

**Table 1:** Cause of admission of rescued birds with age information.

Admission Cause			Age		Number (%)			
Category	Subcategory	Specific Cause	Y *	A **				
Natural	Orphaned	Injured	54	0	54	(3.7)	56	219 (15.0)
		Congenital abnormality	2	0	2	(0.1)		
	Malnutrition	Exhaustion/emaciation	43	45	88	(6.0)	88	
	Infection	Bacteria	2	3	5	(0.3)	66	
		Virus	17	34	51	(3.5)		
		Parasite	4	6	10	(0.7)		
	Attacked	Other birds	2	7	9	(0.6)	9	
Anthropogenic	Orphaned	Unnecessary rescue	344	0	344	(23.5)	344	1016 (69.4)
	Attacked	Cat/dog	2	29	31	(2.1)	31	
	Collision	Window	3	303	306	(20.9)	316	
		Power line/electric shock	0	10	10	(0.7)		
	Trapped indoors	Trapped in a building/farm	48	43	91	(6.2)	110	
		Breeding at the rooftop	16	3	19	(1.3)		
	Injured by human-made structures	Mouse glue trap	3	38	41	(2.8)	104	
		Tied to a string/fishing line	0	19	19	(1.3)		
		Trapped in a net	0	18	18	(1.2)		
		Stranded in a hole/water canal	7	4	11	(0.8)		
		Fishhook	0	6	6	(0.4)		
		Stuck in windows/signboards	1	4	5	(0.3)		
		Mower	0	2	2	(0.1)		
		Oil/contaminated	0	1	1	(0.1)		
		Wrongly landed	0	1	1	(0.1)		
	Traffic accident	Hit by car/train	2	70	72	(4.9)	72	
	Poaching	Gunshot	0	16	16	(1.1)	20	
		Trap	0	4	4	(0.3)		
	Toxicosis	Poisoning	0	19	19	(1.3)	19	
Others	Unknown	Trauma from unknown reason	7	137	144	(9.8)	225	229 (15.6)
		Undiagnosed	4	77	81	(5.5)		
	Other	Confiscated protected species	0	4	4	(0.3)	4	
Total			561	903	1464	(100)	1464 (100)	

* Y, young birds (from eggs to fledgling). ** A, adults, including juveniles.

**Table 2:** Admission causes of top 30 avian species admitted with more than 10 birds.

Common Name	Orp	Mln	Inf	Att	Cll	Trp	Inj	TA	Pch	Tox	Ukn	Total
Oriental Turtle Dove	20%	2%	36%	13%	16%	2%	0%	2%	1%	0%	8%	146
Indian Spot-billed Duck	60%	1%	0%	0%	4%	26%	7%	0%	1%	0%	2%	138
Common Kestrel	21%	17%	4%	0%	12%	3%	13%	6%	1%	0%	23%	125
Feral Pigeon	28%	5%	5%	5%	17%	9%	10%	4%	0%	0%	16%	96
Oriental Magpie	49%	8%	1%	5%	14%	2%	5%	2%	0%	0%	14%	87
Brown-eared Bulbul	17%	8%	0%	3%	32%	5%	6%	3%	0%	5%	22%	65
Eurasian Tree Sparrow	52%	8%	0%	0%	15%	3%	8%	3%	0%	0%	10%	61
Northern Boobook	8%	3%	0%	5%	53%	2%	2%	12%	0%	0%	15%	60
Mandarin Duck	48%	0%	0%	0%	0%	48%	0%	2%	0%	0%	2%	52
Eurasian Eagle-Owl	13%	6%	4%	0%	8%	4%	21%	17%	8%	2%	17%	48
Common Pheasant	81%	0%	0%	0%	3%	3%	8%	3%	0%	0%	3%	37
Oriental Scops Owl	28%	0%	0%	6%	28%	6%	8%	3%	0%	0%	22%	36
Grey Heron	10%	23%	0%	0%	3%	6%	16%	3%	10%	0%	29%	31
Eurasian Goshawk	0%	10%	0%	0%	50%	0%	0%	7%	7%	7%	20%	30
Intermediate Egret	36%	14%	0%	0%	23%	0%	0%	5%	0%	0%	23%	22
Japanese Tit	41%	0%	0%	0%	14%	9%	23%	0%	0%	0%	14%	22
Western Cattle Egret	15%	20%	0%	0%	15%	5%	0%	5%	0%	0%	40%	20
Azure-winged Magpie	50%	11%	6%	0%	11%	0%	17%	0%	0%	0%	6%	18
Eastern Buzzard	0%	6%	0%	0%	6%	0%	17%	22%	11%	28%	11%	18
Oriental Dollarbird	28%	0%	0%	0%	22%	0%	0%	11%	0%	0%	39%	18
Cinereous Vulture	0%	19%	0%	0%	0%	0%	0%	6%	6%	44%	25%	16
Black-tailed Gull	0%	7%	0%	7%	20%	0%	40%	0%	0%	0%	27%	15
Barn Swallow	64%	0%	0%	0%	21%	14%	0%	0%	0%	0%	0%	14
Daurian Redstart	57%	7%	0%	0%	7%	7%	21%	0%	0%	0%	0%	14
Great Egret	0%	0%	0%	0%	14%	0%	0%	7%	7%	0%	71%	14
Gray-headed Woodpecker	0%	0%	0%	0%	69%	8%	8%	0%	0%	0%	15%	13
Japanese Scops Owl	15%	0%	0%	0%	54%	0%	0%	15%	0%	0%	15%	13
White’s Thrush	8%	0%	0%	8%	69%	8%	0%	0%	0%	0%	8%	13
Little Egret	9%	27%	0%	0%	18%	0%	0%	9%	0%	0%	36%	11
Eurasian Sparrowhawk	0%	0%	0%	0%	30%	0%	0%	10%	10%	0%	50%	10

Orp: orphaned, Inf: infection, Mln: malnutrition, Att: attacked, Cll: collision, Trp: trapped indoors, Inj: injured by human-made structures, TA: traffic accident, Pch: poaching, Tox: toxicosis, Ukn: unknown.

**Table 3:** Clinical findings and diagnoses of examined birds by category.

Category	Subcategory	Description	No.	
Circulatory	Cardiac	Injury/myocarditis	13	209 (4.4%)
	Hematologic	Anemia	131	
	Hematologic	Leukocytosis	60	
	Hematologic	Leukopenia	5	
Digestive	Bile duct	Inflammation/injury	4	255 (5.4%)
	Cloaca	Prolapse/injury/inflammation	10	
	Crop	Rupture/ingluvitis	19	
	Esophagus	Injury/esophagitis	9	
	Hepatic	Hepatitis/injury	145	
	Intestines	Enteritis/injury	28	
	Oral	Trauma/inflammation	10	
	Pancreatic	Inflammation/injury	6	
	Stomach	Gastritis/injury	24	
General	Circulation	Dehydration	534	916 (19.2%)
	Circulation	Exhaustion	182	
	Circulation	Hypothermia	54	
	Circulation	Shock	38	
	Malnutrition	Emaciation	108	
Infection	Bacteria	Bacteremia/sepsis	5	374 (7.9%)
	Bacteria	*Campylobacter jejuni*	3	
	Bacteria	Local infection (mainly *Enterococcus*, *Staphylococcus*, Enterobacteriaceae)	55	
	Bacteria	*Mycobacterium*	1	
	Bacteria	*Mycoplasma*	15	
	Parasite	Lice/tick/mite/louse fly	104	
	Parasite	Nematode/trematode/cestode/protozoa	80	
	Fungus	*Aspergillus* spp.	1	
	Parasite	*Haemoproteus* spp./*Plasmodium* spp.	50	
	Virus	Avian influenza	2	
	Virus	Poxvirus	58	
Integumentary	Feather	Contamination	43	961 (20.2%)
	Feather	Damage	18	
	Feather	Loss	51	
	Foot	Bumble foot	5	
	Skin	Dermatitis	5	
	Skin	Foreign body/granuloma	8	
	Skin	Laceration/abrasion/penetration/incision/degloving/hematoma/loss	311	
	Skin	Myiasis	24	
	Skin	Necrosis	39	
	Skin	Vesicle/papule/nodule/abscess	47	
	Subcutaneous	Hemorrhage/edema/emphysema	410	
Musculoskeletal	Amputated	Leg > digit/talon > wing > beak	19	923 (19.4%)
	Fracture	Body: Vertebrae > coracoid > keel bone > clavicle > beak > rib > pelvis > scapula > skull > mandible > maxilla	152	
	Fracture	Leg: tibiotarsus > tarsometatarsus > femur > digit/talon	66	
	Fracture	Wing: humerus > ulna > radius > carpometacarpus > digit	238	
	Infection	Myelitis/periostitis/osteolysis	6	
	Joints	Ankylosis	9	
	Joints	Arthritis	6	
	Luxation	Elbow > shoulder > wrist > coracoid > hock > acetabulum > digit > stifle	49	
	Malunion	Wing	3	
	Muscular	Injury/laceration/incision	330	
	Necrosis	Wing tip syndrome	3	
	Old fracture	Wing > leg > body	35	
	Skeletal	Metabolic bone disease	7	
Nervous	Brain	Traumatic brain injury/inflammation/hemorrhage	299	419 (8.8%)
	Peripheral	Injury (wing = spinal cord > leg)	103	
	Toxicosis	Pesticide, unknown	17	
Respiratory	Air sac	Rupture/airsacculitis	35	171 (3.6%)
	Bronchial	Inflammation/injury	3	
	Nasal	Hemorrhage/rhinitis/sinusitis	50	
	Pulmonary	Injury/pneumonia/infection	80	
	Tracheal	Tracheitis/injury	3	
Sensory	Ocular	Blepharitis	3	358 (7.5%)
	Ocular	Cataract	8	
	Ocular	Conjunctivitis	17	
	Ocular	Corneal edema/bullous keratopathy	6	
	Ocular	Follicular conjunctivitis/laceration of the third eyelid	4	
	Ocular	Glaucoma	1	
	Ocular	Intraocular hemorrhage, uveitis, corneal abrasion/ulcer, retinal laceration/detachment, vitreous prolapse, iris rupture, lens luxation, asteroid hyalosis	294	
	Ocular	Orbital edema	7	
	Ocular	Rupture/phthisis bulbi	8	
	Otic	Trauma/inflammation	10	
Urinary	Renal	Acute kidney insufficiency/inflammation/injury	164	167 (3.5%)
	Renal	Gout	3	
Total				4753 (100%)

## Data Availability

The original contributions presented in this study are included in the article/Supplementary Materials; further inquiries can be directed to the corresponding author.

## Supplementary Materials

The following supporting information can be downloaded at: https://www.mdpi.com/article/10.3390/ani14142071/s1, Table S1: Demographic and age information of rescued wild birds in this study; Table S2: Monthly analysis of admission causes; Table S3: Admission causes categorized by trauma and inflammatory conditions.
