# Supplementary material for: Morbidity of Rescued Wild Birds by Admission Causes in the Republic of Korea
Source: Animals (Basel). 2024 Jul 15;14(14):2071. doi: 10.3390/ani14142071 (PMC11273627; doi:10.3390/ani14142071)
Supplement: Supplementary file 1 [file animals-14-02071-s001.zip › animals-3039393-supplementary.pdf]

Supplementary.

Table S1. Demographic and age information of rescued wild birds in this study

Order	Family	Scientific name	Common name	No.	%	Age	
						Y*	A**
Anseriformes	Anatidae	*Aix galericulata*	Mandarin Duck	52	3.6	48	4
		Anas acuta	Northern Pintail	1	0.1	0	1
		Anas crecca	Eurasian Teal	5	0.3	0	5
		Anas formosa	Baikal Teal	1	0.1	0	1
		Anas platyrhynchos	Mallard	4	0.3	2	2
		Anas poecilorhyncha	Indian Spot-billed Duck	138	9.4	122	16
		Anas strepera	Gadwall	2	0.1	0	2
		Anser albifrons	Greater White-fronted Goose	4	0.3	0	4
		Anser cygnoides domesticus	Domestic goose	1	0.1	0	1
		Anser serrirostris	Tundra Bean Goose	4	0.3	0	4
		Cygnus cygnus	Whooper Swan	4	0.3	1	3
Accipitriformes	Accipitridae	*Accipiter gentilis*	Eurasian Goshawk	30	2.0	0	30
		Accipiter gularis	Japanese Sparrowhawk	5	0.3	1	4
		Accipiter nisus	Eurasian Sparrowhawk	10	0.7	0	10
		Accipiter soloensis	Chinese Sparrowhawk	1	0.1	0	1
		Aegypius monachus	Cinereous Vulture	16	1.1	0	16
		Buteo japonicus	Eastern Buzzard	18	1.2	0	18
		Circus cyaneus	Hen Harrier	1	0.1	0	1
		Pernis ptilorhynchus	Crested Honey Buzzard	1	0.1	0	1
Bucerotiformes	Upupidae	*Upupa epops*	Eurasian Hoopoe	5	0.3	0	5
Caprimulgiformes	Caprimulgidae	*Caprimulgus jotaka*	Grey Nightjar	4	0.3	0	4
Charadriiformes	Alcidae	*Synthliboramphus antiquus*	Ancient Murrelet	1	0.1	0	1
	Charadriidae	*Charadrius alexandrinus*	Kentish Plover	1	0.1	1	0
		Vanellus vanellus	Northern Lapwing	1	0.1	0	1
	Laridae	*Larus crassirostris*	Black-tailed Gull	15	1.0	0	15
		Larus vegae	Vega Gull	2	0.1	0	2
	Scolopacidae	*Gallinago gallinago*	Common Snipe	1	0.1	0	1
		Limnodromus scolopaceus	Long-billed Dowitcher	1	0.1	0	1
		Limosa lapponica	Bar-tailed Godwit	1	0.1	0	1
		Numenius arquata	Eurasian Curlew	2	0.1	0	2
		Numenius phaeopus	Eurasian Whimbrel	2	0.1	0	2
		Scolopax rusticola	Eurasian Woodcock	6	0.4	0	6
Columbiformes	Columbidae	*Columba livia domestica*	Feral Pigeon	96	6.6	37	59
		Streptopelia orientalis	Oriental Turtle Dove	146	10.0	52	94
Coraciiformes	Alcedinidae	*Alcedo atthis*	Common Kingfisher	9	0.6	1	8
		Halcyon coromanda	Ruddy Kingfisher	2	0.1	0	2
	Coraciidae	*Eurystomus orientalis*	Oriental Dollarbird	18	1.2	5	13
Cuculiformes	Cuculidae	*Cuculus canorus*	Common Cuckoo	4	0.3	1	3
		Cuculus optatus	Oriental Cuckoo	2	0.1	0	2
Falconiformes	Falconidae	*Falco peregrinus*	Peregrine Falcon	5	0.3	0	5
		Falco subbuteo	Eurasian Hobby	5	0.3	0	5

Order	Family	Scientific name	Common name	No.	%	Age	
						Y*	A**
		Falco tinnunculus	Common Kestrel	125	8.5	40	85
Galliformes	Phasianidae	*Coturnix japonica*	Japanese Quail	1	0.1	0	1
		Lophura swinhoii	Swinhoe's Pheasant	4	0.3	0	4
		Phasianus colchicus	Common Pheasant	37	2.5	30	7
Gaviiformes	Gaviidae	*Gavia arctica*	Black-throated Loon	1	0.1	0	1
Gruiformes	Rallidae	*Fulica atra*	Eurasian Coot	6	0.4	0	6
Passeriformes	Aegithalidae	*Aegithalos caudatus*	Long-tailed Tit	1	0.1	0	1
	Alaudidae	*Alauda arvensis*	Eurasian Skylark	1	0.1	0	1
	Bombycillidae	*Bombycilla japonica*	Japanese Waxwing	1	0.1	0	1
	Corvidae	*Cyanopica cyanus*	Azure-winged Magpie	18	1.2	13	5
		Garrulus glandarius	Eurasian Jay	4	0.3	3	1
		Pica serica	Oriental Magpie	87	5.9	51	36
	Fringillidae	*Chloris sinica*	Grey-capped Greenfinch	3	0.2	2	1
		Coccothraustes coccothraustes	Hawfinch	2	0.1	0	2
		Eophona migratoria	Chinese Grosbeak	7	0.5	0	7
		Fringilla montifringilla	Brambling	1	0.1	0	1
		Spinus spinus	Eurasian Siskin	1	0.1	0	1
	Hirundinidae	*Cecropis daurica*	Red-rumped Swallow	1	0.1	0	1
		Hirundo rustica	Barn Swallow	14	1.0	9	5
	Laniidae	*Lanius bucephalus*	Bull-headed Shrike	1	0.1	0	1
		Lanius collurio	Red-backed Shrike	1	0.1	1	0
	Motacillidae	*Anthus hodgsoni*	Olive-backed Pipit	1	0.1	0	1
		Motacilla alba	White Wagtail	1	0.1	0	1
	Muscicapidae	*Cyanoptila cyanomelana*	Blue-and-white Flycatcher	2	0.1	0	2
		Ficedula mugimaki	Mugimaki Flycatcher	1	0.1	0	1
		Ficedula zanthopygia	Yellow-rumped Flycatcher	1	0.1	0	1
		Phoenicurus auroreus	Daurian Redstart	14	1.0	9	5
	Oriolidae	*Oriolus chinensis*	Black-naped Oriole	6	0.4	1	5
	Paridae	*Parus minor*	Japanese Tit	22	1.5	10	12
	Passeridae	*Passer montanus*	Eurasian Tree Sparrow	61	4.2	36	25
	Phylloscopidae	*Phylloscopus examinandus*	Kamchatka Leaf Warbler	5	0.3	0	5
	Pycnonotidae	*Hypsipetes amaurotis*	Brown-eared Bulbul	65	4.4	19	46
	Sittidae	*Sitta europaea*	Eurasian Nuthatch	2	0.1	0	2
	Sturnidae	*Spodiopsar cineraceus*	White-cheeked Starling	4	0.3	4	0
	Sylviidae	*Acrocephalus orientalis*	Oriental Reed Warbler	1	0.1	0	1
		Phylloscopus borealis	Arctic Warbler	1	0.1	0	1
		Phylloscopus tenellipes	Pale-legged Leaf Warbler	1	0.1	0	1
		Urosphena squameiceps	Asian Stubtail	4	0.3	0	4
	Paradoxornithidae	*Suthora webbiana*	Vinous-throated Parrotbill	4	0.3	0	4
	Turdidae	*Turdus hortulorum*	Grey-backed Thrush	3	0.2	0	3
		Turdus obscurus	Eyebrowed Thrush	1	0.1	0	1
		Turdus pallidus	Pale Thrush	3	0.2	2	1
		Zoothera aurea	White's Thrush	13	0.9	1	12
Pelecaniformes	Ardeidae	*Ardea alba alba*	Great Egret	1	0.1	0	1
		Ardea alba modesta	Great Egret	14	1.0	0	14
		Ardea cinerea	Grey Heron	31	2.1	6	25
		Ardea intermedia	Intermediate Egret	22	1.5	9	13
		Bubulcus ibis	Western Cattle Egret	20	1.4	9	11

**Order**	**Family**	**Scientific name**	**Common name**	**No.**	**%**	**Age**	
						**Y***	**A****
		Butorides striata	Striated Heron	5	0.3	2	3
		Egretta garzetta	Little Egret	11	0.8	2	9
		Ixobrychus eurhythmus	Von Schrenck's Bittern	4	0.3	0	4
		Nycticorax nycticorax	Black-crowned Night Heron	3	0.2	2	1
	Phalacrocoracidae	*Phalacrocorax carbo*	Great Cormorant	3	0.2	0	3
	Sulidae	*Sula sula*	Red-footed Booby	1	0.1	0	1
Piciformes	Picidae	*Dendrocopos leucotos*	White-backed Woodpecker	4	0.3	0	4
		Dendrocopos major	Great Spotted Woodpecker	2	0.1	0	2
		Picus canus	Grey-headed Woodpecker	13	0.9	0	13
Podicipediformes	Podicipedidae	*Podiceps cristatus*	Great Crested Grebe	3	0.2	0	3
		Tachybaptus ruficollis	Little Grebe	2	0.1	1	1
Strigiformes	Strigidae	*Asio otus*	Long-eared Owl	3	0.2	0	3
		Bubo bubo kiautschensis	Eurasian Eagle-Owl	48	3.2	6	42
		Ninox japonica	Northern Boobook	60	4.1	6	54
		Otus semitorques	Japanese Scops Owl	13	0.9	2	11
		Otus sunia	Oriental Scops Owl	36	2.5	11	25
		Strix nivicolum	Himalayan Owl	5	0.3	3	2
Total				1464	100	561	903

Y*: young birds (from eggs to fledgling), A**: adults including juveniles

Table S2. Monthly analysis of admission causes. The records of all birds from January 2019 to February 2021 were included.

**Category**	**Specific cause**	**Month**												**Total**
		**1**	**2**	**3**	**4**	**5**	**6**	**7**	**8**	**9**	**10**	**11**	**12**	
Orphaned	Unnecessary rescue			3	23	77	111	78	34	13		5		344
	Injured		1	1	6	18	9	13	3	2		1		54
	Congenital abnormality					1	1							2
Malnutrition	Exhaustion/emaciation	8	1	1	1	11	32	15	10	2	3	1	3	88
Infection	Bacteria				1		1	1		1	1			5
	Virus						1	17	11	6	13	3		51
	Parasite					1	2	2	1	1	1	2		10
Attacked	Cat/dog attacks	2	2	4	7	1	3	1	1	3	2	3	2	31
	Other birds			1	4	1		2					1	9
Collision	Window	31	29	16	23	25	27	23	34	35	28	21	14	306
	Power line/electric shock	2	2		2					2		1	1	10
Trapped indoors	Trapped in a building/farm	6	3	2	5	19	33	11	2	6	1	3		91
	Breeding at the rooftop						16					3		19
Injured by human-made structures	Mouse glue trap	3		1	4	3	4	9	3	9	3		2	41
	Tied to a string/fishing line	1	3	2		1	2	3	2	3	2			19
	Trapped in a net	2	4				2	2	1	2		2	3	18
	Stranded in a hole/water canal		1	1		7			2					11
	Fishhook	1				1		1		1	1	1		6
	Stuck in windows/signboards	1			1		2					1		5
	Mower						2							2
	Oil/contaminated					1								1
	Wrongly landed		1											1
Traffic accident	Hit by car/train	9	4	3	1	12	7	6	8	4	7	4	7	72
Poaching	Gunshot	2	4	1		1		1		1	2		4	16
	Trap	1			1		1	1						4
Toxicosis	Poisoning	6	2	6								2	3	19
Unknown	Trauma with unknown reason	10	10	4	7	21	16	19	30	11	3	1	12	144
	Undiagnosed	9	4	7	4	8	4	10	14	7	6	4	4	81
Other	Confiscated protected species	4												4
Total		98	71	53	90	209	276	215	156	109	73	58	56	1464

Table S3. Admission causes categorized by trauma and inflammatory

Category	Group	Specific cause	N	Rate	Category
Trauma (n=750, 51.2%)	Orphaned	Injured	54	3.7%	Inflammatory (n=835, 57%)
	Attacked	Cat/dog	31	2.1%	
		Other birds	9	0.6%	
	Collision	Window	306	20.9%	
		Power line/electric shock	10	0.7%	
	Injured by human-made structures	Mouse glue trap	41	2.8%	
		Tied to a string/fishing line	19	1.3%	
		Trapped in a net	18	1.2%	
		Stranded in a hole/water canal	11	0.8%	
		Fishhook	6	0.4%	
		Stuck between windows/signboards	5	0.3%	
		Grass cutter	2	0.1%	
		Oil/contaminated	1	0.1%	
		Wrongly landed	1	0.1%	
	Traffic accident	Hit by car/train	72	4.9%	
	Poaching	Gun shot	16	1.1%	
		Trap	4	0.3%	
	Unknown	Trauma with unknown reason	144	9.8%	
Non-trauma (n=714, 48.8%)	Infection	Bacteria	5	0.3%	
		Virus	51	3.5%	
		Parasite	10	0.7%	
	Toxicosis	Poisoning	19	1.3%	
	Orphaned	Unnecessary rescue	344	23.5%	Non-inflammatory (n=629, 43%)
		Congenital abnormality	2	0.1%	
	Malnutrition	Exhaustion/cachexia	88	6.0%	
	Trapped indoors	Trapped in a building/farm	91	6.2%	
		Breeding at the rooftop	19	1.3%	
	Unknown	Undiagnosed	81	5.5%	
	Other	Confiscated protected species	4	0.3%	
Total			1464	100%	
